# On the Role of Molecular Conformation of the 8-Oxoguanine Lesion in Damaged DNA Processing by Polymerases

**DOI:** 10.1021/acs.jcim.2c01430

**Published:** 2023-02-24

**Authors:** Inacrist Geronimo, Pietro Vidossich, Marco De Vivo

**Affiliations:** Laboratory of Molecular Modelling & Drug Discovery, Istituto Italiano di Tecnologia, Via Morego 30, Genoa 16163, Italy

## Abstract

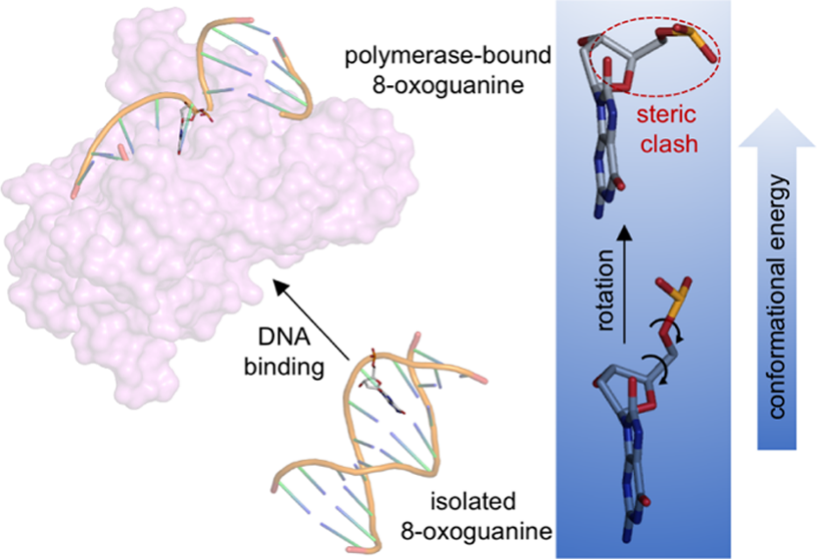

A common and insidious DNA damage is 8-oxoguanine (8OG), bypassed with low catalytic efficiency and high error frequency by polymerases (Pols) during DNA replication. This is a fundamental process with far-reaching implications in cell function and diseases. However, the molecular determinants of how 8OG exactly affects the catalytic efficiency of Pols remain largely unclear. By examining ternary deoxycytidine triphosphate/DNA/Pol complexes containing the 8OG damage, we found that 8OG consistently adopts different conformations when bound to Pols, compared to when in isolated DNA. Equilibrium molecular dynamics and metadynamics free energy calculations quantified that 8OG is in the lowest energy conformation in isolated DNA. In contrast, 8OG adopts high-energy conformations often characterized by intramolecular steric repulsion when bound to Pols. We show that the 8OG conformation can be regulated by mutating Pol residues interacting with the 8OG phosphate group. These findings propose the 8OG conformation as a factor in Pol-mediated processing of damaged DNA.

## Introduction

**Scheme 1:**
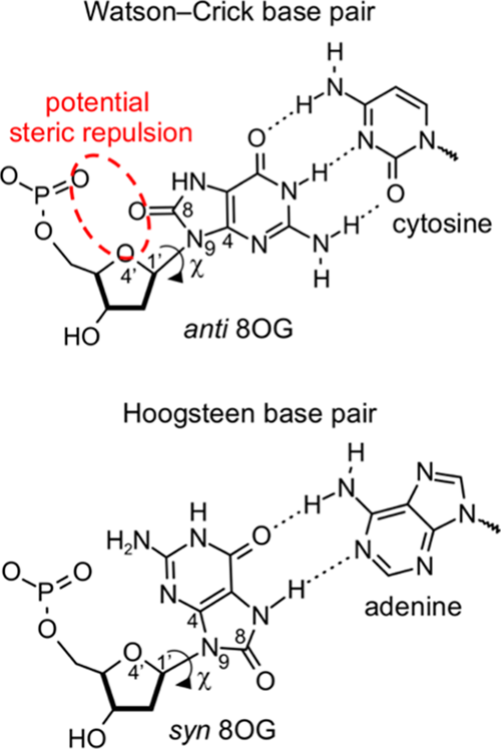
*Anti* and *Syn* Conformations of the Glycosyl Torsion Angle [χ(O4′–C1′–N9–C4)] of 8OG Leading to Watson–Crick and Hoogsteen Base Pairing, Respectively

Oxidative DNA damage is caused by reactive oxygen species produced during normal cellular processes or generated by external agents such as ionizing radiation, UV light, or chemotherapy drugs.^1^ Among the nucleobases, guanine is the most susceptible to oxidation because it has the lowest redox potential.^2^ One of its most common oxidation products is 8-oxo-7,8-dihydro-2′-guanosine (8OG), which is oxidized at the C8 position (Scheme 1). Notably, 8OG is commonly used as a biomarker of oxidative DNA damage to assess the risk of cancer and age-related diseases.^3−5^

8OG can be incorporated into genomic DNA by either direct oxidation of deoxyguanosine (dG) in the DNA or addition of oxidized dG triphosphate from the nucleotide pool during DNA synthesis by DNA polymerases (Pols),^5^ the enzymes responsible for DNA replication and repair.^6−8^ However, 8OG is highly mutagenic because it can form a Hoogsteen base pair with the wrong base, adenine, via a switch of the glycosyl torsion angle χ from the anti to the syn conformation (Scheme 1).^9^ The 8OG:A base pair can lead to transversion mutation in genomic DNA in the subsequent round of replication (e.g., G:C → T:A, with 8OG as the templating base).^10^ Indeed, the 8OG:A base pair can escape proofreading given its geometrical similarity to the Watson–Crick base pair T:A.^11−13^

However, how does 8OG exactly affect the catalytic efficiency and error frequency of Pols? From experiments,^13−21^ Pols are known to incorporate the correct nucleotide, deoxycytidine triphosphate (dCTP), opposite 8OG with lower catalytic efficiency (*k*_cat_/*K*_M_) and higher error frequency compared with those for incorporation opposite dG. For example, the Pol I fragment from *Bacillus stearothermophilus*, which has the lowest reported error frequency for the incorporation of dCTP versus deoxyadenosine triphosphate (dATP) opposite dG (approximately one error per 10^7^ incorporations), has one of the highest error frequencies when the templating base is 8OG (essentially an error at each incorporation).^13^ The catalytic efficiency for dCTP incorporation also decreases dramatically from 500 min^–1^ μM^–1^ opposite dG to 0.13 min^–1^ μM^–1^ opposite 8OG.

In this regard, the high error frequency of Pols is often attributed to the propensity of 8OG to switch to the syn conformation to avoid an intramolecular steric clash between the C8 oxo group (O8) and the sugar–phosphate backbone, which can occur in the anti conformation (Scheme 1).^22,23^ This conformational switch facilitates the binding of an incoming dATP and the formation of a stable Hoogsteen base pair.^11^ On the other hand, the cause of the reduced catalytic efficiency for dCTP incorporation is less clear because structural comparison of the matched 8OG:dCTP and dG:dCTP ternary complex crystal structures of various Pols^12,14−17,22−26^ indicates that the oxidation of dG to 8OG does not alter the two-metal active site geometry^27,28^ of the Michaelis–Menten complex (Figure S1). As a matter of fact, the isolated 8OG-damaged DNA duplex is nearly structurally indistinguishable from the undamaged one because 8OG adopts the same conformation as dG.^29^ It is therefore still largely unclear how 8OG affects the catalytic efficiency of Pols.

By inspecting kinetically characterized ternary dCTP/DNA/Pol complexes comprising the 8OG damage, we noticed that 8OG adopts different specific molecular conformations, which can be defined according to the phosphate group orientation with respect to the base and sugar groups. Using well-tempered metadynamics^30^ and equilibrium molecular dynamics (MD) simulations, we could energetically quantify that Pol-bound 8OG adopts high-energy conformations, compared to 8OG in isolated DNA. We propose the 8OG conformation as a factor in Pol-mediated processing of damaged DNA.

## Results and Discussion

### *Anti* 8OG Has a Different Conformation in dCTP/DNA/Pol Ternary Complexes than in Isolated DNA

We analyzed the ternary dCTP/DNA/Pol complexes comprising the 8OG damage. We considered only Pol complexes for which kinetic data are available to look for a structure–function relationship. In this way, we could survey eight crystal structures of ternary Pol complexes, with the incoming dCTP facing the template 8OG. These include the Pol from bacteriophage T7 (T7 Pol, A-family),^12^ Pol from bacteriophage RB69 (RB69 Pol, B-family),^14^ human Pol μ (X-Family),^15^ human Pol λ (X-family),^16^ human Pol β (X-family),^24^*Sulfolobus solfataricus* Pol IV Dpo4 (Y-family),^25^ and human Pol η (Y-family),^17^ as well as the bifunctional human DNA primase/polymerase (PrimPol).^26^ Notably, most Pols are in a reactive configuration,^31,32^ wherein the O3′ atom of the terminal primer residue is coordinated to the metal at site A and aligned with the P_α_ atom of the incoming dCTP for nucleophilic attack (Figure S1). Even in cases where the site A metal and/or O3′ atom is missing (T7 Pol, RB69 Pol, Pol λ, and Dpo4), the enzyme is in the closed conformation required for catalysis. The overall organization of the active site residues is similar to those in the corresponding undamaged dG:dCTP ternary complexes. This structural similarity suggests that the oxidation of dG to 8OG does not impede the formation of a Michaelis–Menten complex.

**Scheme 2:**
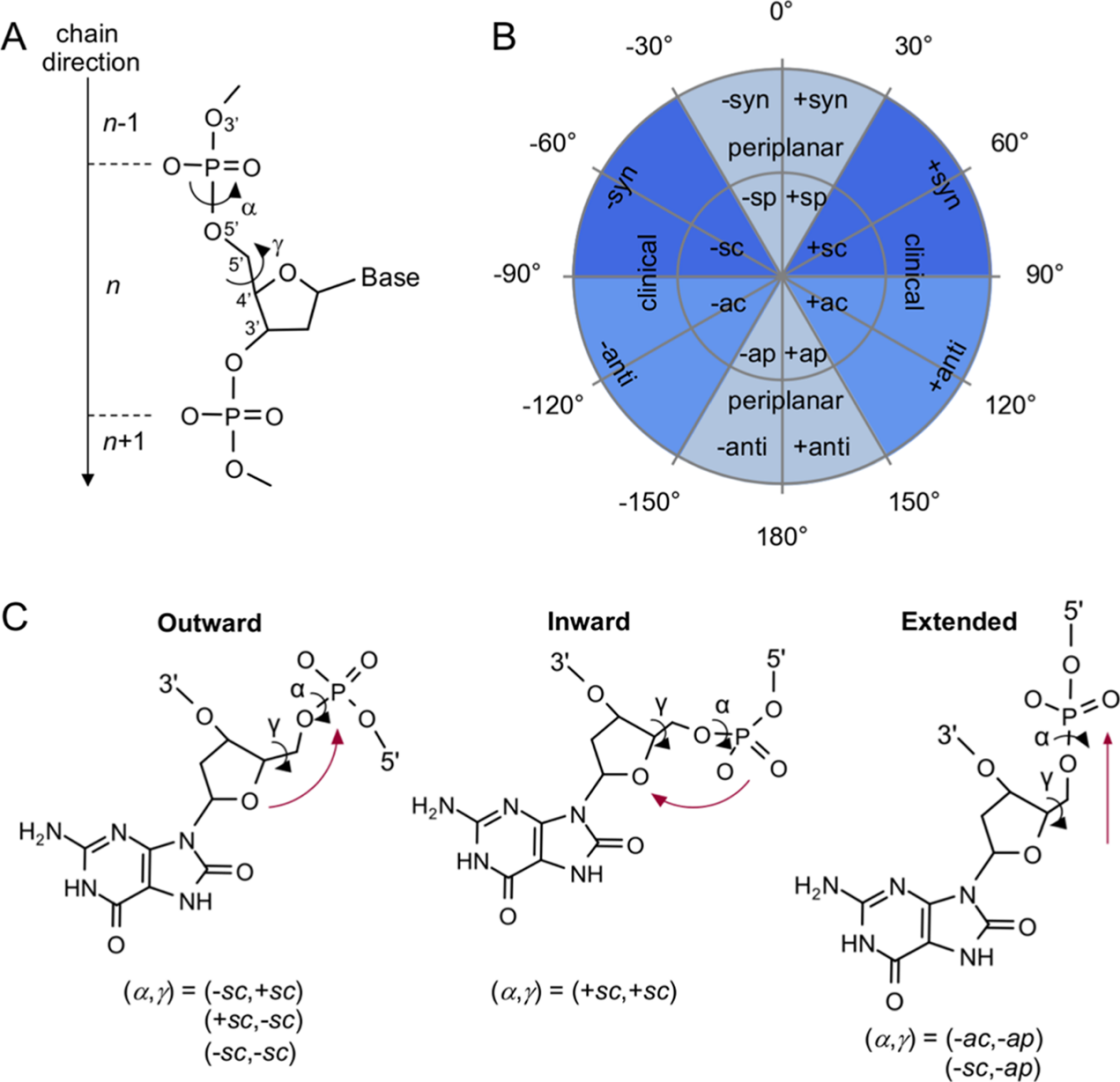
(A) α[(*n* – 1)O3′–P–O5′–C5′] and γ(O5′–C5′–C4′–C3′) Torsion Angles of a Nucleotide; (B) Klyne–Prelog Nomenclature for Torsion Angle Conformation; and (C) Backbone Conformations of 8OG Classified according to the Phosphate Group Orientation with Respect to the Sugar and Base Groups

Importantly, we noticed that in all such Pol complexes, the template 8OG always adopts the *anti* glycosyl torsion angle conformation (hereafter referred to as *anti* 8OG). The highly correlated α and γ torsion angles (Scheme 2A) define the contact between the base, sugar, and phosphate groups of a nucleotide.^33,34^ These torsion angles were therefore chosen as a metric to measure the geometrical differences of the specific *anti* 8OG conformation in Pol-bound DNA compared to that in isolated B-DNA. We used the Klyne–Prelog nomenclature for torsion angle conformations,^35,36^ namely, ±*synclinal* (±*sc*), ±*anticlinal* (±*ac*), ±*synperiplanar* (±*sp*), and ±*antiperiplanar* (±*ap*), to classify each *anti* 8OG conformation (see Scheme 2B for torsion angle ranges). In particular, we refer to the “outward” conformation of the 8OG backbone when its α, γ torsion angle conformation is (−*sc*, +*sc*), (−*sc*, −*sc*), or (+*sc*, −*sc*). This conformation reflects a phosphate group rotated away from the sugar and base groups (Scheme 2C). In contrast, we refer to the “extended” conformation of the 8OG backbone when its α, γ torsion angle conformation is (−*ac*, −*ap*) or (−*sc*, −*ap*). In this conformation, the phosphate backbone is stretched out owing to a ≈90° bend in the template strand at the point of entry in the Pol active site (Scheme 2C). Finally, we refer to the “inward” conformation of the 8OG backbone when its α, γ torsion angle conformation is (+*sc*, +*sc*). Here, there is close contact between the O8 atom and one phosphate O atom (Scheme 2C).

**Figure 1:**
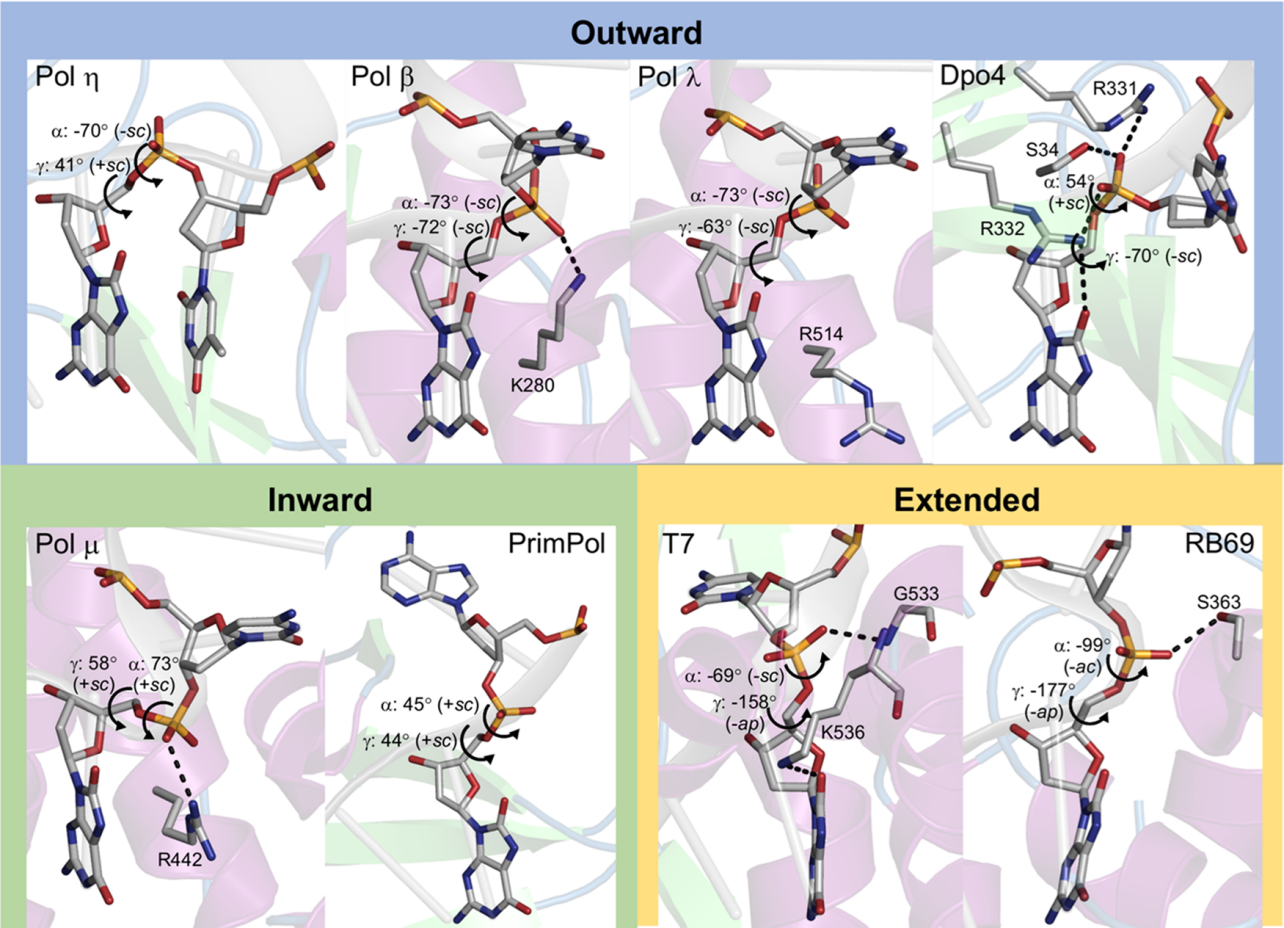
Crystal structures of the matched *anti* 8OG:deoxycytidine triphosphate (dCTP) ternary complexes of human DNA polymerase (Pol) η (PDB ID 4O3P(17)), human Pol β (PDB ID 4RPX(24)), human Pol λ (PDB ID 5IIJ(16)), *Sulfolobus solfataricus* Pol IV Dpo4 (PDB ID 2ASD(25)), human Pol μ (PDB ID 6P1P(15)), human DNA primase/polymerase (PrimPol, PDB ID 7JK1(26)), Pol from bacteriophage T7 (PDB ID 1TK0(12)), and Pol from bacteriophage RB69 (PDB ID 1Q9Y(14)). The Pols are grouped into outward, inward, and extended conformations according to the 8OG backbone conformation (see Scheme 2). The α[(*n* – 1)O3′–P–O5′–C5′] and γ(O5′–C5′–C4′–C3′) torsion angles of *anti* 8OG with the corresponding conformations are indicated.

In isolated B-DNA, the α, γ conformation is always outward, specifically (−*sc*, +*sc*). In this specific conformation, there are no close contacts between the base, sugar, and phosphate groups of *anti* 8OG. On the other hand, in our dataset of ternary Pol complexes, only the *anti* 8OG in Pol η exhibits this specific α, γ conformation (Figure 1). The *anti* 8OG in Pol β, Pol λ, and Dpo4 also has an outward conformation, but instead of (−*sc*, +*sc*), the specific α, γ conformations are (−*sc*, −*sc*), (−*sc*, −*sc*), and (+*sc*, −*sc*), respectively. In RB69 and T7 Pols, the *anti* 8OG conformation is extended, where the O8 atom is near the ribose O4′ atom (O8–O4′ distances of 2.5 and 2.7 Å, respectively, Figure 1). *Anti* 8OG has the inward conformation in Pol μ and PrimPol. In the case of the former, the O8 atom is near a phosphate oxygen (a O8–OP distance of 3.1 Å, Figure 1). Thus, we note that the 8OG backbone conformation in the ternary complexes of our dataset differs from the conformation adopted in the isolated B-DNA. That is, the 8OG backbone always adopts an outward (−*sc*, +*sc*) conformation in the isolated DNA, while it adopts inward, extended, or other outward conformations when the 8OG-damaged DNA is embedded in ternary Pol complexes, with the exception of Pol η. Importantly, we also note that, unlike the outward conformation in the isolated DNA, only the inward and extended conformations can generate intramolecular steric repulsion. Such a clash is caused by the O8 oxygen located close to the ribose or phosphate oxygen only when inward and extended conformations are adopted. Taken together, these unprecedented structural observations point out a different conformational equilibrium of the DNA and 8OG when bound to the Pol enzyme, as compared to 8OG in isolated DNA.

### *Anti* 8OG Adopts High-Energy Conformations When Bound to Pols Mostly Due to Interactions of Its Phosphate Group with Surrounding Pol Residues

**Figure 2:**
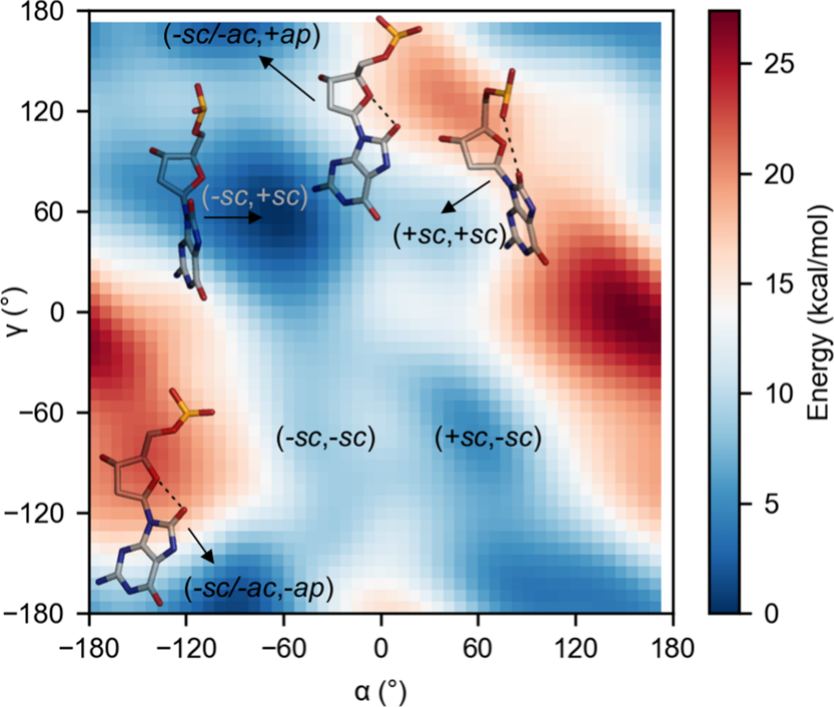
FES of *anti* 8OG in isolated DNA in the phase space of the α[(*n* – 1)O3′–P–O5′–C5′] and γ(O5′–C5′–C4′–C3′) torsion angles. Representative structures of selected conformational states are also shown. The dashed lines represent steric repulsion between O8 and a ribose or phosphate oxygen. Estimated errors in free energies are shown in Figure S2.

Based on the structural evidence reported above, we calculated the relative energies of the different conformations of *anti* 8OG. Toward this end, the isolated 8OG-damaged B-DNA (PDB ID 183D(29)) was used as the model to exclude environmental effects (e.g., H-bonding with protein residues), which may affect the conformational equilibrium. The free energy surface (FES) of *anti* 8OG in the phase space of the α and γ torsion angles (Scheme 2A) was mapped by performing a well-tempered metadynamics simulation. The outward (−*sc*, +*sc*) conformation is the lowest energy conformation (Figure 2). Notably, this conformation is seen only in the ternary complex of Pol η. Relative to this conformation, the conformations adopted by *anti* 8OG in all the other Pol active sites are higher in energy (Figure 2 and Table S1). The other outward conformations, (+*sc*, −*sc*) in Dpo4 and (−*sc*, −*sc*) in Pol β and Pol λ, are 5.0 and 8.1 kcal/mol higher in energy than (−*sc*, +*sc*) even if there is no steric clash between the base, sugar, and phosphate groups. The highest energy conformation by 9.0 kcal/mol is the inward conformation, (+*sc*, +*sc*) in Pol μ and PrimPol, wherein there is a steric clash between O8 and a phosphate O atom. On the other hand, despite the close contact between the O8 and O4′ atoms in extended conformations [(−*ac*, −*ap*) in RB69 Pol and (−*sc*, −*ap*) in T7 Pol], the energy is only 1.9 kcal/mol higher than the outward (−*sc*, +*sc*) conformation. Thus, the backbone conformations adopted by *anti* 8OG in the Pol active site are higher in energy than the outward (−*sc*, +*sc*) conformation adopted in isolated DNA, even when a steric clash between O8 and either the ribose or phosphate oxygen is absent.

To look for a possible role of the enzyme and explain the different conformational preferences of 8OG in the ternary complexes, we performed 1 μs unbiased MD simulations of the matched 8OG:dCTP complexes of two X-family Pols, Pol μ (PDB ID 6P1P(15)) and Pol β (PDB ID 4RPX(24)). The protein and DNA backbone root-mean-square deviations (RMSDs) (Figure S3) show that both complexes remain structurally stable throughout the MD simulations. Moreover, they both maintain a reactive configuration, wherein the nucleophile (O3′ of the terminal primer residue) is coordinated to the site A Mg and aligned with P_α_ of the incoming dCTP (Table S2).

**Figure 3:**
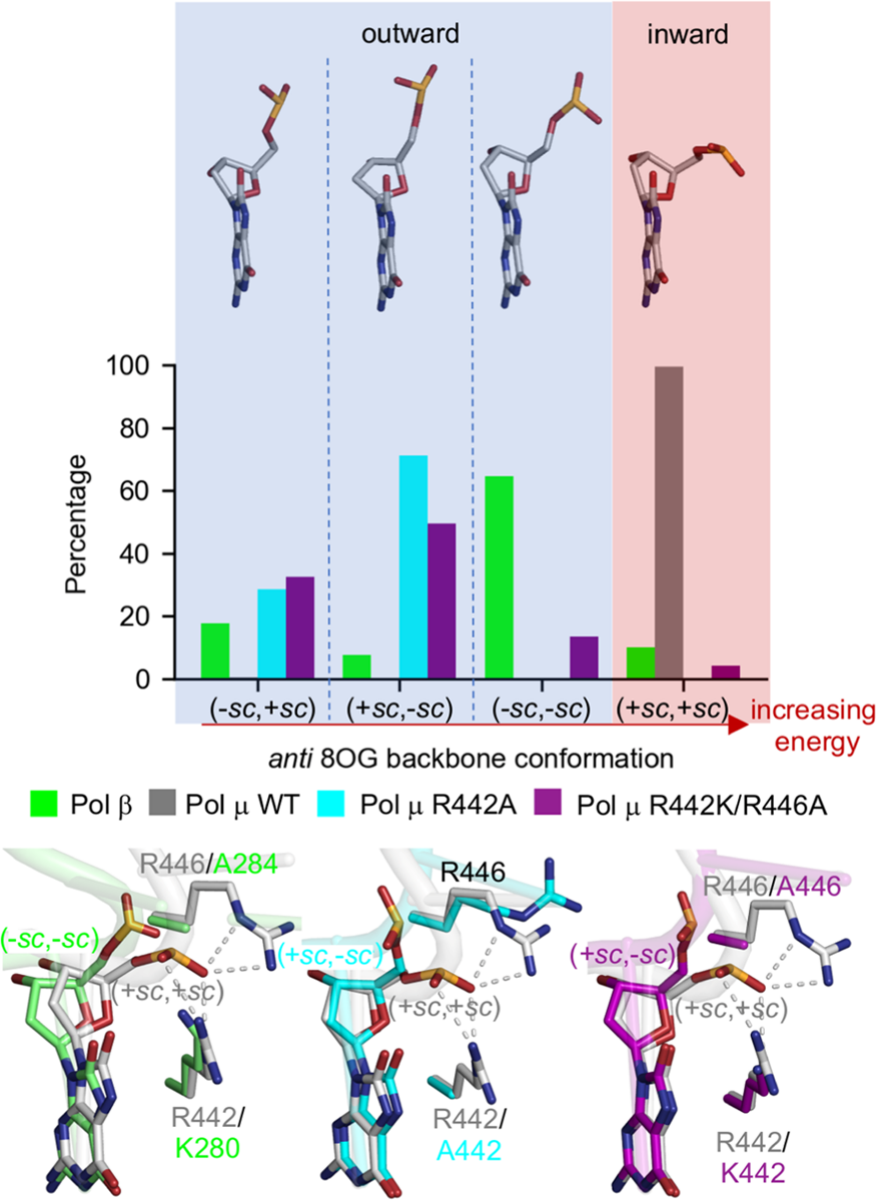
*Anti* 8OG backbone conformations in MD structures of DNA polymerase (Pol) β and WT and mutant (R442A and R442K/R446A) Pol μ. The *anti* 8OG backbone adopts a lower energy outward conformation [(−*sc*, +*sc*), (+*sc*, −*sc*), or (−*sc*, −*sc*)] in Pol β and mutant Pol μ and the high-energy inward conformation (+*sc*, +*sc*) in WT Pol μ. In WT Pol μ, H-bond interactions with R442 and R446 (gray dashed lines) hinder the rotation of the 8OG backbone to a lower energy conformation.

In the crystal structures, *anti* 8OG adopts the high-energy inward conformation (+*sc*, +*sc*) in Pol μ and a lower energy outward conformation (−*sc*, −*sc*) in Pol β. During the MD simulations, *anti* 8OG maintains the inward conformation in Pol μ but samples all the three outward conformations [(−*sc*, −*sc*), (−*sc*, +*sc*), and (+*sc*, −*sc*)], as well as the inward conformation, in Pol β (Figure 3). This difference in the *anti* 8OG backbone flexibility can be partially explained by the stability of the H-bond interaction of the phosphate group of 8OG. In Pol μ, the phosphate group is H-bonded to R442 and R446. These interactions are maintained during the MD simulations with average H-bond occupancies of 79 and 38%, respectively. These stable interactions restrict the 8OG backbone to the inward conformation. In contrast, in Pol β, the H-bond between the phosphate group and more flexible K280 observed in the crystal structure is broken during the MD simulations, allowing the 8OG backbone to switch between inward and outward conformations. Additionally, we note that the rotation of the 8OG backbone to outward conformations in Pol μ appears to be sterically hindered by R446 in the N-helix. Notably, this residue corresponds to an alanine in Pol β (A284), where such conformational switch of 8OG occurs.

To verify the roles of R442 and R446 in the *anti* 8OG backbone conformational preference in Pol μ, we also performed 1 μs unbiased MD simulations of Pol μ mutants, namely, R442A and R442K/R446A. For the R442A mutant, the 8OG backbone was initially modeled in the inward conformation as in wild-type (WT) Pol μ. On the other hand, for the R442K/R446A mutant, which is supposed to mimic the protein environment of *anti* 8OG in Pol β, the 8OG backbone was initially modeled in the outward (−*sc*, −*sc*) conformation as in Pol β. The R442A and R442K/R446A mutants are structurally similar to the WT (protein backbone RMSDs of 0.7 and 1.0 Å, respectively, and DNA backbone RMSDs of 0.8 and 1.1 Å, respectively, Figure S3) and maintain a reactive active site configuration (Table S2). Based on these simulations, *anti* 8OG switches from the high-energy inward conformation in WT Pol μ to low-energy outward conformations [(+*sc*, −*sc*) and (−*sc*, +*sc*)] in the R442A and R442K/R446A mutants (Figure 3).

One of these conformations, (−*sc*, +*sc*), is the lowest energy backbone conformation that is also exhibited by *anti* 8OG in isolated DNA and in Pol η. On the other hand, the R442K/R446A double mutation fails to stabilize the outward (−*sc*, −*sc*) conformation observed in Pol β, which indicates that other residues and factors are likely to affect the 8OG backbone conformation. The residues around 8OG and its adjacent base downstream are not well conserved in X-family Pols. Interestingly, all these residues are positively charged in Pol μ (R442, R446, R449, K450, and R181), which is not the case in Pol β (K280, A284, L287, E288, and K41, respectively, Figure S4) and Pol λ (R514, A518, K521, T522, and K281, respectively). Thus, the 8OG backbone conformation seems to mainly depend on the interactions of the 8OG phosphate group with the surrounding protein residues. In the case of Pol μ, 8OG is surrounded by multiple positively charged residues that restrict the rotation of its backbone. Eliminating the interaction with some of these residues allows *anti* 8OG to switch from the high-energy inward conformation to low-energy outward conformations.

**Figure 4:**
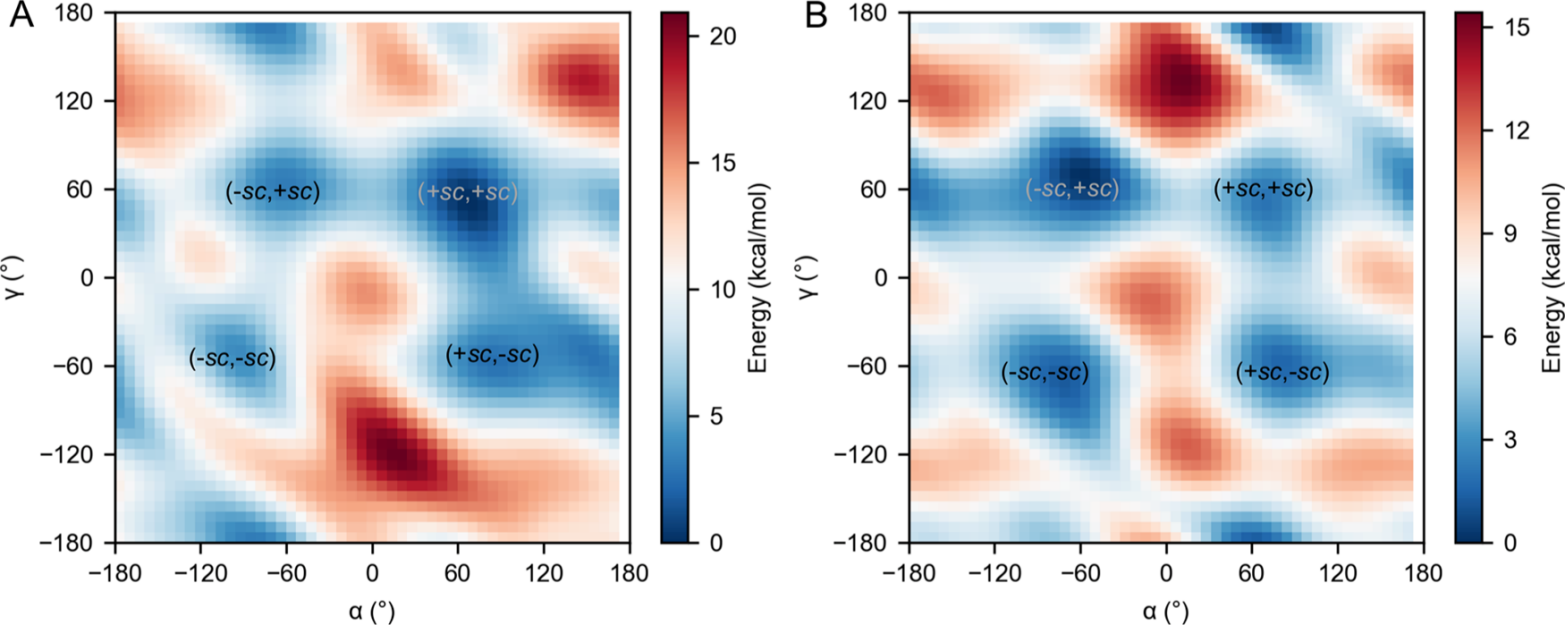
FES of *anti* 8OG in (A) polymerase (Pol) μ-bound and (B) Pol β-bound DNA in the phase space of the α[(*n* – 1)O3′–P–O5′–C5′] and γ(O5′–C5′–C4′–C3′) torsion angles. The main conformational states are indicated. The protein environment alters the conformational equilibrium so that the lowest energy conformation is different in the two Pols (+*sc*, +*sc* in Pol μ and −*sc*, +*sc* in Pol β). Estimated errors in free energies are shown in Figure S2.

Subsequently, we investigated the effect of protein interactions on the FES of *anti* 8OG by performing well-tempered metadynamics simulations of the 8OG:dCTP/DNA/Pol complexes of Pol μ and Pol β. As shown in Figure 4 and Table S1, unlike the case in isolated DNA, the lowest energy conformation of *anti* 8OG is (+*sc*, +*sc*) in Pol μ-bound DNA, consistent with the fact that only this conformation was observed in the crystal structure (PDB ID 6P1P) and during the unbiased MD simulations (Figure 3). On the other hand, for Pol β-bound DNA, although the (−*sc*, −*sc*) conformation is the one adopted in the crystal structure (PDB ID 4RPX), the free energy calculations showed that it is close in energy to the (−*sc*, +*sc*), (+*sc*, −*sc*), and (+*sc*, +*sc*) conformations. All four conformations were observed during the unbiased MD simulations (Figure 3). Thus, these results confirm that environmental effects can indeed alter the conformational equilibrium of *anti* 8OG in the Pol active site. Notably, despite the steric repulsion between O8 and the phosphate group of *anti* 8OG, the (+*sc*, +*sc*) conformation has the lowest energy in Pol μ-bound DNA, presumably owing to the stabilizing effect of the hydrogen bonds with R442 and R446. Such interactions are broken when *anti* 8OG shifts from (+*sc*, +*sc*) to another conformation, resulting in an increase in energy.

## Conclusions

In this study, we characterized the conformation of Pol-bound *anti* 8OG and investigated its potential functional significance in the replication of 8OG-damaged DNA by Pols. The *anti* 8OG conformation was classified into outward, inward, and extended conformations according to the phosphate group orientation with respect to the base and sugar groups. We showed that the *anti* 8OG in isolated DNA is in the lowest energy outward conformation. In comparison, the inward and extended conformations exhibited by Pol-bound *anti* 8OG are higher in energy. This seems mostly due to the steric clash between the O8 atom and a phosphate or ribose oxygen. We showed that the 8OG molecular conformation can be modulated by mutating protein residues interacting with the 8OG phosphate group. These results could therefore aid in the design of more efficient and processive Pols for the amplification of damaged DNA.

## Methods

### Well-Tempered Metadynamics

The FESs of *anti* 8OG in isolated, Pol μ-bound, and Pol β-bound DNA were obtained by well-tempered metadynamics. System preparation and equilibration by unbiased MD are described below (for Pol-bound DNA) and in the Supporting Information (for isolated DNA). For the well-tempered metadynamics simulations, the α and γ backbone torsion angles were used as the collective variables (Scheme 2A). Gaussians with a height of 0.239 kcal mol^–1^ and a width of 0.35 rad (for both α and γ) were deposited every picosecond, and the bias factor was set to 10. The simulations were run for 100–200 ns (see convergence plots in Figure S5) using GROMACS 2020.5^37^ patched with PLUMED 2.7.1.^38,39^ The ensemble average free energy ⟨*A*⟩_ξ_ over all configurations with ξ(α,γ) = ξ (see Scheme 2B for the intervals in the α,γ configurational space defining each backbone conformation) was calculated using the following equation
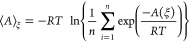
1where *A* is the free energy from the free energy profile generated from the metadynamics data, ξ is the reaction coordinate (i.e., collective variable), *R* is the gas constant (1.987 × 10^–3^ kcal K^–1^ mol^–1^), *T* is the temperature (310 K), and *n* is the number of frames with ξ(α,γ) = ξ. The free energy difference Δ*F* between two configurations is simply the difference between their ⟨*A*⟩_ξ_ values (for more details, see refs (40) and (41)). The error in free energy was estimated by reweighting the metadynamics simulation and performing block analysis. Conformational potential energies were validated by performing reference quantum chemical calculations of structures taken from the metadynamics simulations, as discussed in the Supporting Information.

### Unbiased MD Simulations

The backbone torsion angle conformations of Pol-bound 8OG were investigated by simulating four ternary Pol complexes: (1) ternary 8OG(*anti*):dCTP/DNA/Pol μ, (2) ternary 8OG(*anti*):dCTP/DNA/Pol μ R442A mutant, (3) ternary 8OG(*anti*):dCTP/DNA/Pol μ R442K/R446A mutant, and (4) ternary 8OG(*anti*):dCTP/DNA/Pol β. Systems (1) to (3) were modeled based on PDB ID 6P1P (1.75 Å)^15^ and system (4) on PDB ID 4RPX (1.90 Å).^24^ The Pol μ crystal structure is that of a truncated catalytic domain (P132–A434), wherein the disordered loop connecting β-strands 4 and 5 (loop 2, P398–P410) has been replaced by Gly410 to improve crystallization.^15,42^ This modification was retained in our models since the deletion has been shown to have no significant effect on the gap-filling activity of Pol μ.^42^ On the other hand, missing residues in loop 1 (C369–F385) and the N-terminal end of the catalytic domain were modeled using Modeller 10.1.^43^ The procedures for system preparation and simulation, including the active site charges and force fields used, are described in the Supporting Information.^31^ Production simulations were performed for 1 μs for each system (total of 4 μs) using GROMACS 2020.6.^37^ Geometric parameters and H-bond interactions were analyzed using CPPTRAJ.^44^

## Data and Software Availability

PDB files were downloaded from the RCSB Protein Data Bank (https://www.rcsb.org). MODELLER (https://salilab.org/modeller/) was used to add the missing protein residues. GROMACS (https://www.gromacs.org/) and PLUMED (https://www.plumed.org/) were used to perform classical MD and well-tempered metadynamics simulations. CPPTRAJ (http://ambermd.org/) was used for post-MD analyses. Coordinate and free energy files are available from the authors upon request.

## Supporting Information Available

Computational details, correlation analysis, and additional data from unbiased MD and well-tempered metadynamics simulations (PDF)
