# Supplementary material for: On the Role of Molecular Conformation of the 8-Oxoguanine Lesion in Damaged DNA Processing by Polymerases
Source: J Chem Inf Model. 2023 Feb 24;63(5):1521–8. doi: 10.1021/acs.jcim.2c01430 (PMC10015460; doi:10.1021/acs.jcim.2c01430)
Supplement: Supplementary file 1 — ci2c01430_si_001.pdf [file ci2c01430_si_001.pdf]

SUPPORTING INFORMATION

On the Role of Molecular Conformation of the 8-Oxoguanine Lesion in Damaged DNA Processing by Polymerases

Inacrist Geronimo, Pietro Vidossich, and Marco De Vivo*

Laboratory of Molecular Modelling & Drug Discovery, Istituto Italiano di Tecnologia, Via Morego 30, Genoa 16163, Italy

***Corresponding author:**

Marco De Vivo, Email: marco.devivo@iit.it

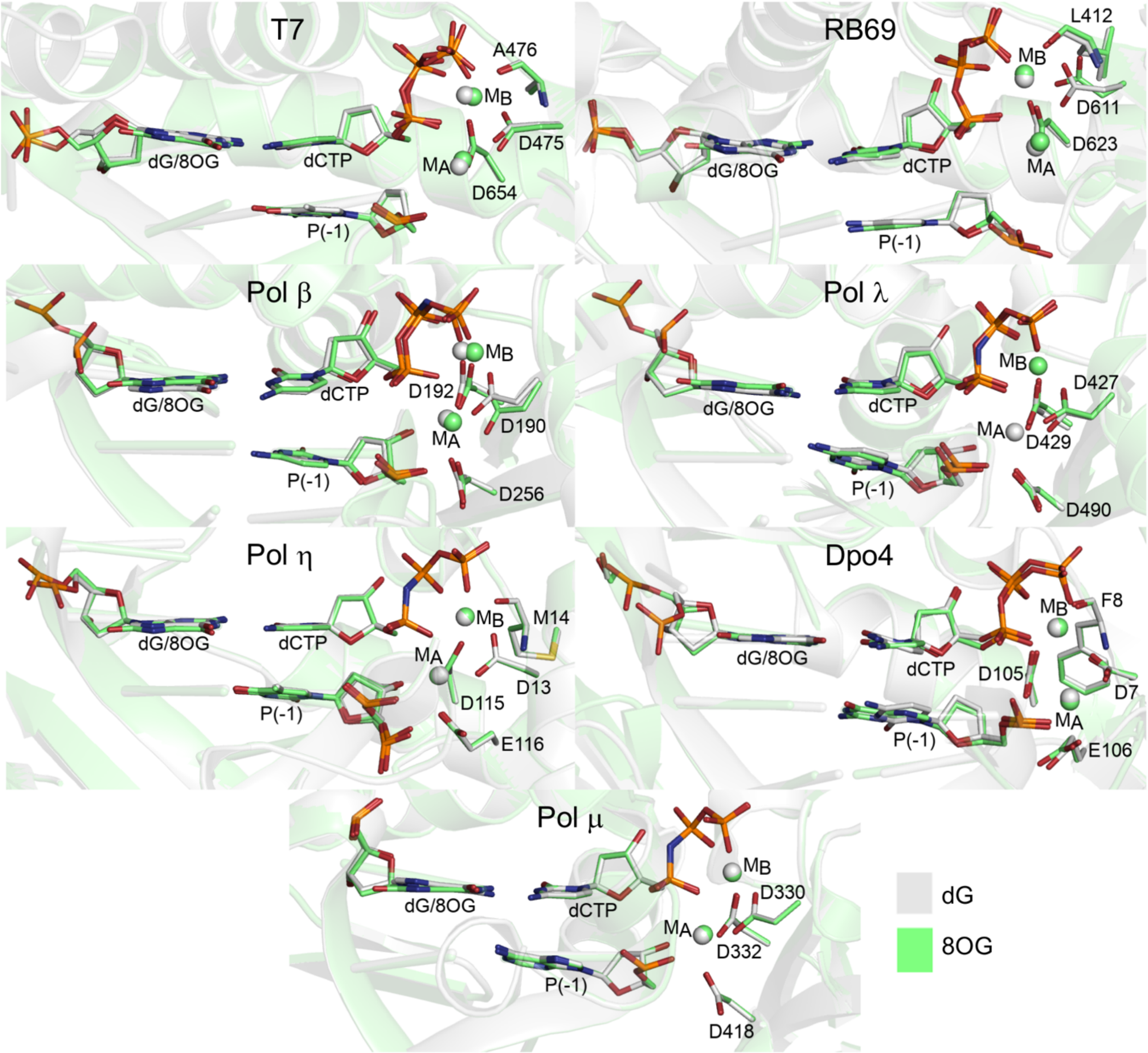

Figure S1. Comparison of DNA polymerase (Pol) crystal structures with deoxyguanosine (dG) or 8-oxo-7,8-dihydro-2′-guanosine (8OG) as the templating base and deoxycytidine triphosphate (dCTP) as the incoming nucleotide. The PDB IDs are listed in Table S3.

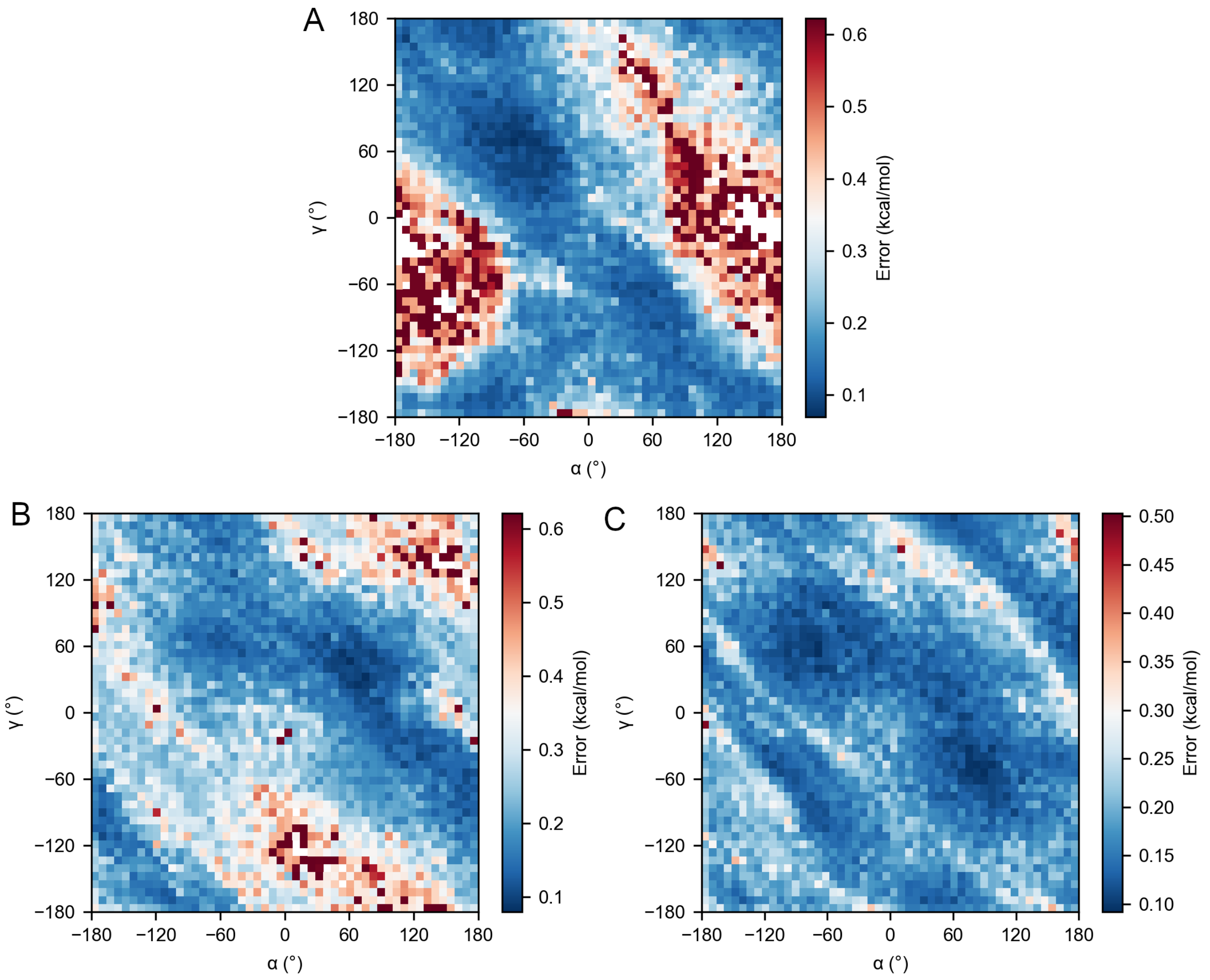

Figure S2. Error estimates of the free energy surfaces of *anti* 8-oxo-7,8-dihydro-2′-guanosine in (A) isolated, (B) polymerase (Pol) μ-bound, and (C) Pol β-bound DNA in the phase space of the α [(n-1)O3′-P-O5′-C5′] and γ(O5′-C5′-C4′-C3′) torsion angles obtained by block analysis (block size of 1000).

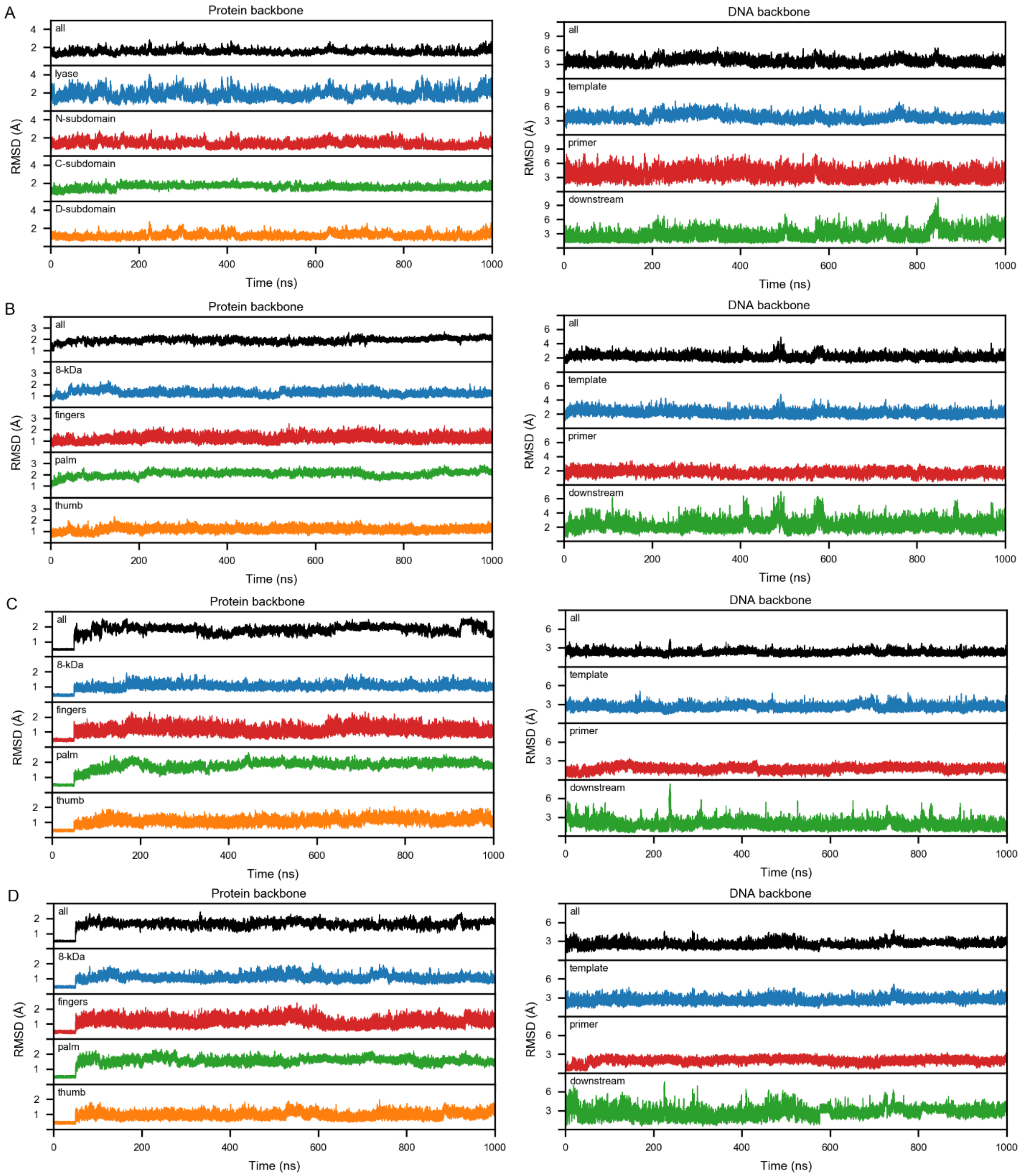

Figure S3. Time evolution of backbone RMSDs (Å) in the matched 8-oxo-7,8-dihydro-2′-guanosine (8OG):deoxycytidine triphosphate (dCTP) complexes of (A) DNA polymerase (Pol) β, (B) wild-type Pol μ, (C) Pol μ R442A mutant, and (D) Pol μ R442K/R446A mutant. For Pol μ, the flexible loop 1 (C369–F385), which was missing from the crystal structure and added by modeling, was excluded from the calculation. For the Pol μ mutants, the protein backbone atoms were restrained during the first 50 ns of simulation.

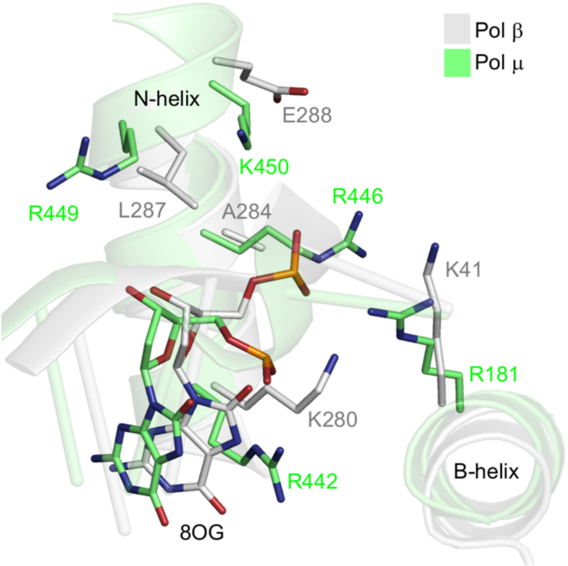

Figure S4. Crystal structures of X-family DNA polymerases μ (PDB ID 6P1P[1]) and β (PDB ID 4RPX[2]) with 8-oxo-7,8-dihydro-2′-guanosine (8OG). The residues around 8OG are not conserved.

Table S1. Relative energies (Δ*F*) of α,γ conformations of 8-oxo-7,8-dihydro-2′-guanosine in different systems calculated by well-tempered metadynamics.

α,γ	Δ*F* (kcal/mol)		
	Isolated DNA	Pol μ-bound DNA	Pol β-bound DNA
-sc,+sc [a]	0.00	0.00	0.00
-ac,-ap	1.87	0.96	4.94
-sc,-ap	1.93	1.42	5.08
+sc,-sc	4.96	-0.40	1.59
-sc,-sc	8.11	1.54	1.15
+sc,+sc	8.95	-2.98	1.93

[a] Common reference point.

Table S2. Geometric parameters from MD simulations of the matched 8-oxo-7,8-dihydro-2′-guanosine (8OG):deoxycytidine triphosphate (dCTP) ternary complexes of DNA polymerase (Pol) μ and Pol β.

Geometric parameter [a]	Pol μ			Pol β
	Wild-type	R442A	R442K/ R446A	
MgA-D330(D190)@OD2	1.96 ± 0.05	1.97 ± 0.05	1.97 ± 0.05	1.96 ± 0.05
MgA-D332(D192)@OD1	1.99 ± 0.05	1.99 ± 0.05	1.98 ± 0.05	1.98 ± 0.05
MgA-D418(D256)@OD2	1.98 ± 0.05	1.98 ± 0.05	1.98 ± 0.05	1.97 ± 0.05
MgA-P(-1)@O3'	2.2 ± 0.1	2.2 ± 0.1	2.2 ± 0.1	2.2 ± 0.1
MgA-dCTP@OA	2.2 ± 0.1	2.2 ± 0.2	2.2 ± 0.2	2.4 ± 0.2
MgA-H_2O	2.07 ± 0.06	2.07 ± 0.06	2.07 ± 0.06	2.07 ± 0.06
MgA-MgB	3.9 ± 0.1	3.9 ± 0.1	3.9 ± 0.1	3.8 ± 0.1
MgB-D330(D190)@OD1	1.99 ± 0.05	1.98 ± 0.05	1.97 ± 0.05	1.97 ± 0.05
MgB-D332(D192)@OD2	2.01 ± 0.06	2.00 ± 0.05	2.01 ± 0.06	2.01 ± 0.06
MgB-dCTP@OA	2.3 ± 0.2	2.4 ± 0.2	2.4 ± 0.2	2.2 ± 0.2
MgB-dCTP@OB	2.00 ± 0.05	2.00 ± 0.05	1.99 ± 0.05	1.99 ± 0.05
MgB-dCTP@OG	1.95 ± 0.05	1.94 ± 0.04	1.94 ± 0.04	1.94 ± 0.04
MgB-H_2O	2.10 ± 0.07	2.09 ± 0.07	2.10 ± 0.07	2.10 ± 0.08
P(-1)@O3'-dCTP@PA	3.6 ± 0.2	3.7 ± 0.2	3.7 ± 0.2	3.3 ± 0.1
P(-1)@O3'-dCTP@PA-dCTP@OA	164 ± 4	164 ± 4	163 ± 5	168 ± 6

[a] Distances are given in Å and angles in degrees. P(-1) is the terminal primer residue (i.e., nucleophile). The residues in parenthesis are those of Pol β.

Table S3. α,γ Conformations of 8-oxo-7,8-dihydro-2′-guanosine (8OG) and deoxyguanosine (dG) in crystal structures of DNA polymerase ternary complexes.

Pol	8OG			dG		
	PDB ID	Ref.	α,γ	PDB ID	Ref.	α,γ
Pol β	4RPX	2	*-sc,-sc*	5UGP	3	*+sc,+sc*
Pol λ	5IIJ	4	*-sc,-sc*	2PFP	5	*+sc,+sc*
Pol μ	6P1P	1	*+sc,+sc*	6P1V	1	*+sc,+sc*
Pol η	4O3P	6	*-sc,+sc*	4O3N	6	*-sc,+sc*
Dpo4	2ASD	7	*+sc,-sc*[a]	4QW8	8	*-ac,+sc*
T7	1TK0	9	*-sc,-ap*	1T8E	9	*+ac,-sc*
RB69	1Q9Y	10	*-ac,-ap*	3NCI	11	*+ap,+ap*
PrimPol	7JK1	12	*+sc,+sc*[a]	-	-	-

[a] There are two molecules in the crystal with the same α,γ conformation.

COMPUTATIONAL DETAILS

System preparation of isolated 8-oxo-7,8-dihydro-2′-guanosine (8OG)-damaged DNA. Initial coordinates of the isolated DNA duplex with *anti* 8OG were taken from the crystal structure with PDB ID 183D (1.60 Å).[13] The system was solvated in a rhombic dodecahedral box of TIP3P[14] water, with a buffer distance of 12 Å between each wall and the closest atom in each direction. Na^+ and Cl^- ions were added to neutralize the system and achieve an ionic concentration of 25 mM. The parmbsc1[15] force field was used to describe the non-bonded and bonded interactions. To derive charges for 8OG, the *anti* and *syn* conformations of 8OG were first optimized at the MP2/6-31G* level, and the corresponding electrostatic potentials were subsequently calculated at the HF/6-31G* level using Gaussian 09.[16] Finally, a multi-conformational restrained electrostatic potential (RESP)[17,18] fitting over the two 8OG conformations was performed using antechamber.[19] Only the charges of the purine, C1′, and H1′ atoms were derived, while the rest of the atoms were constrained to have the same charges as those in undamaged deoxyguanosine (dG) (Table S4). Prior to the well-tempered metadynamics simulations, the system was minimized, heated, and equilibrated for 100 ns following the protocol described in our previous paper.[20]

Table S4. Derived partial charges of 8-oxo-7,8-dihydro-2′-guanosine (8OG).[a]

Atom name	Atom type	Charge	Atom name	Atom type	Charge
N9	N*	-0.004566	H1	H	0.348606
C8	C	0.408236	C2	CA	0.717325
O8	O	-0.490468	N2	N2	-0.811768
N7	NA	-0.548021	H21	H	0.372421
H7	H	0.398226	H22	H	0.372421
C5	CB	0.007740	N3	NC	-0.529776
C6	C	0.609879	C4	CB	0.106197
O6	O	-0.625336	C1′	CT	0.543252
N1	NA	-0.576626	H1′	H2	-0.176142

[a] For the other atoms, the atom types and charges are the same as those of deoxyguanosine (dG).

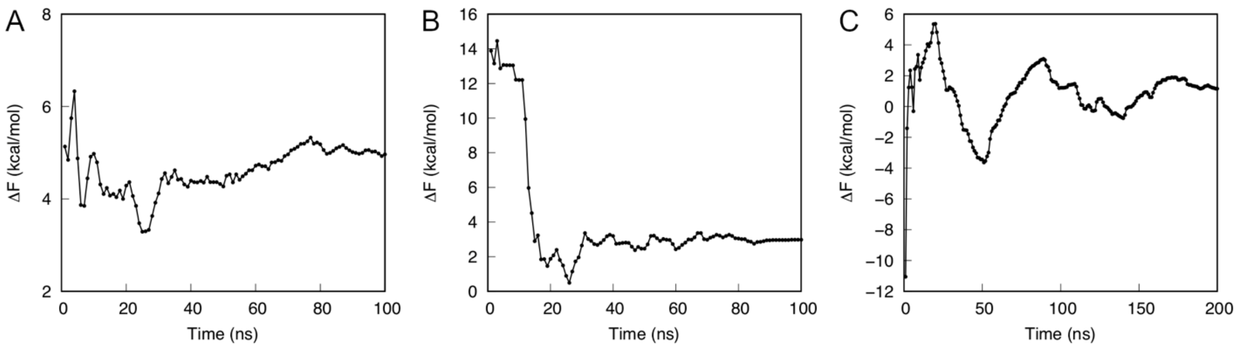

Figure S5. Time evolution of the free energy difference (ΔF) between selected α,γ conformations of 8-oxo-7,8-dihydro-2′-guanosine in (A) isolated (+*sc*,-*sc* and -*sc*,+*sc*), (B) polymerase (Pol) μ-bound (-*sc*,+*sc* and +*sc*,+*sc*), and (C) Pol β-bound DNA (-*sc*,-*sc* and -*sc*,+*sc*).

Quantum chemical calculations of 8OG models. Five snapshots, corresponding to different 8OG backbone conformations, were extracted from the well-tempered metadynamics simulation of the isolated 8OG-damaged DNA duplex. 8OG was extracted from each snapshot, and its valence at the P and O3′ ends were saturated with -OH and -H, respectively. The fragment underwent energy minimization at both the molecular mechanics (MM) and quantum mechanics (QM) levels. Restraints were imposed on the dihedral angles highlighted in Figure S6 to keep the 8OG conformation close to that in the duplex and avoid the formation of intramolecular H-bonds. QM calculations were performed with Gaussian 09[16] using the B3LYP hybrid functional[21,22] and 6-311+G(d,p) basis set. The calculated data (Table S5) indicate a fair correspondence between the MM and QM structures (heavy atom RMSD <0.5 Å) and good agreement between the MM and QM energies (mean absolute error of 1.8 kcal/mol).

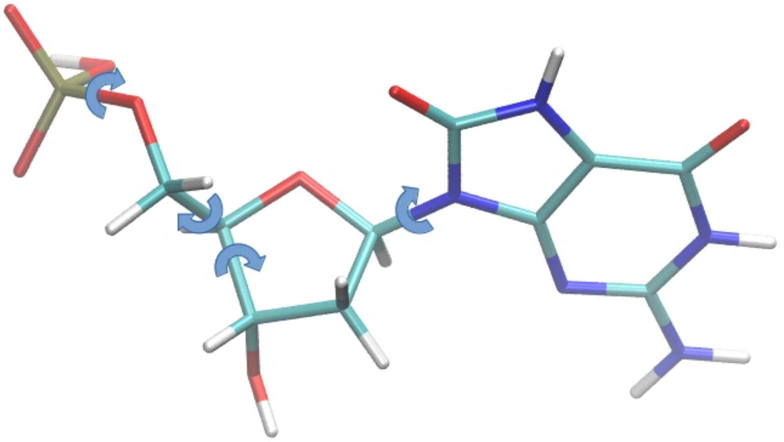

Figure S6. 8-Oxo-7,8-dihydro-2′-guanosine model used to investigate the accuracy of the molecular mechanics force field compared to quantum chemical calculations. The arrows highlight the dihedral angles restrained during energy minimization.

Table S5. Energies (E, kcal/mol) computed at the molecular mechanical (MM) and quantum mechanical (QM) levels and root-mean-square deviation (RMSD) between the MM and QM geometries of selected conformations of 8-oxo-7,8-dihydro-2′-guanosine.

frame	α (°)	γ (°)	E(MM)	E(QM)	RMSD (Å)
1	-96.6	-179.1	0.0	0.0	0.36
2	31.3	61.1	10.9	9.2	0.30
3	-64.8	56.4	-4.5	-7.5	0.49
4	-81.3	-175.7	1.9	2.9	0.23
5	-69.2	163.4	4.1	5.7	0.21

System preparation of 8OG:deoxycytidine triphosphate (dCTP)/DNA/Pol complexes. The systems were solvated in a rhombic dodecahedral box of TIP3P[14] water, with a buffer distance of 12 Å between each wall and the closest atom in each direction. They were then neutralized with K^+, and additional Mg^{2+}, K^+, and Cl^- ions were added to achieve ionic concentrations of 50 mM KCl and 2.5 mM $MgCl_2$. The protein and DNA were described using the AMBER14ffSB[23] and parmbsc1[15] force fields, respectively. The metal active site was treated using a flexible nonbonded approach to allow structural rearrangements at the active site during the MD simulations.[24] The charges of Mg^{2+} and ligating O atoms were taken from our previous

study,[20] and the van der Waals parameters of Mg^{2+} from Allnér et al.[25] dCTP charges were taken from RESP ESP charge DDataBase.[26]

Unbiased molecular dynamics simulations of 8OG:dCTP/DNA/Pol complexes. Minimization was performed for 1000 steps using the steepest descent algorithm, followed by the conjugate gradient algorithm until the maximum force was less than 100 kJ mol^{-1} nm^{-1}. The systems were heated to 310 K for 100 ps in the NVT ensemble and equilibrated for another 500 ps in the NPT ensemble with all heavy atoms restrained. Production simulations were run for 1 μs for each system. Constant temperature was maintained using Langevin dynamics[27] with a time coupling constant of 2 ps. A constant pressure of 1 bar was maintained using the Berendsen algorithm[28] during equilibration, and the Parrinello-Rahman algorithm[29] during production with a time coupling constant of 2 ps. Periodic boundary conditions were applied, and long-range electrostatic interactions were calculated using the particle mesh Ewald method[30] with a real-space cut-off of 12 Å. Bonds with hydrogen were constrained using the LINCS algorithm,[31] which allowed a time step of 2 fs.

References

(1) Kaminski, A. M.; Chiruvella, K. K.; Ramsden, D. A.; Kunkel, T. A.; Bebenek, K.; Pedersen, L. C. Unexpected Behavior of DNA Polymerase Mu Opposite Template 8-Oxo-7,8-Dihydro-2′-Guanosine. *Nucleic Acids Res.* **2019**, *47*, 9410–9422.

(2) Vyas, R.; Reed, A. J.; Tokarsky, E. J.; Suo, Z. Viewing Human DNA Polymerase β Faithfully and Unfaithfully Bypass an Oxidative Lesion by Time-Dependent Crystallography. *J. Am. Chem. Soc.* **2015**, *137*, 5225–5230.

(3) Shock, D. D.; Freudenthal, B. D.; Beard, W. A.; Wilson, S. H. Modulating the DNA Polymerase β Reaction Equilibrium to Dissect the Reverse Reaction. *Nat. Chem. Biol.* **2017**, *13*, 1074–1080.

(4) Burak, M. J.; Guja, K. E.; Hambardjieva, E.; Derkunt, B.; Garcia-Diaz, M. A Fidelity Mechanism in DNA Polymerase Lambda Promotes Error-Free Bypass of 8-Oxo-DG. *EMBO J.* **2016**, *35*, 2045–2059.

(5) Garcia-Diaz, M.; Bebenek, K.; Krahn, J. M.; Pedersen, L. C.; Kunkel, T. A. Role of the Catalytic Metal during Polymerization by DNA Polymerase Lambda. *DNA Repair* **2007**, *6*, 1333–1340.

(6) Patra, A.; Nagy, L. D.; Zhang, Q.; Su, Y.; Müller, L.; Guengerich, F. P.; Egli, M. Kinetics, Structure, and Mechanism of 8-Oxo-7,8-Dihydro-2′-Deoxyguanosine Bypass by Human DNA Polymerase η. *J. Biol. Chem.* **2014**, *289*, 16867–16882.

(7) Rechkoblit, O.; Malinina, L.; Cheng, Y.; Kuryavyi, V.; Broyde, S.; Geacintov, N. E.; Patel, D. J. Stepwise Translocation of Dpo4 Polymerase during Error-Free Bypass of an OxoG Lesion. *PLoS Biol.* **2006**, *4*, e11.

(8) Gaur, V.; Vyas, R.; Fowler, J. D.; Efthimiopoulos, G.; Feng, J. Y.; Suo, Z. Structural and Kinetic Insights into Binding and Incorporation of L-Nucleotide Analogs by a Y-Family DNA Polymerase. *Nucleic Acids Res.* **2014**, *42*, 9984–9995.

(9) Brieba, L. G.; Eichman, B. F.; Kokoska, R. J.; Doublié, S.; Kunkel, T. A.; Ellenberger, T. Structural Basis for the Dual Coding Potential of 8-Oxoguanosine by a High-Fidelity DNA Polymerase. *EMBO J.* **2004**, *23*, 3452–3461.

(10) Freisinger, E.; Grollman, A. P.; Miller, H.; Kisker, C. Lesion (in)Tolerance Reveals Insights into DNA Replication Fidelity. *EMBO J.* **2004**, *23*, 1494–1505.

(11) Wang, M.; Xia, S.; Blaha, G.; Steitz, T. A.; Konigsberg, W. H.; Wang, J. Insights into Base Selectivity from the 1.8 Å Resolution Structure of an RB69 DNA Polymerase Ternary Complex. *Biochemistry* **2011**, *50*, 581–590.

(12) Rechkoblit, O.; Johnson, R. E.; Gupta, Y. K.; Prakash, L.; Prakash, S.; Aggarwal, A. K. Structural Basis of DNA Synthesis Opposite 8-Oxoguanine by Human PrimPol Primase-Polymerase. *Nat. Commun.* **2021**, *12*, 4020.

(13) Lipscomb, L. A.; Peek, M. E.; Morningstar, M. L.; Verghis, S. M.; Miller, E. M.; Rich, A.; Essigmann, J. M.; Williams, L. D. X-Ray Structure of a DNA Decamer Containing 7,8-Dihydro-8-Oxoguanine. *Proc. Natl. Acad. Sci.* **1995**, *92*, 719–723.

(14) Jorgensen, W. L.; Chandrasekhar, J.; Madura, J. D.; Impey, R. W.; Klein, M. L. Comparison of Simple Potential Functions for Simulating Liquid Water. *J. Chem. Phys.* **1983**, *79*, 926–935.

(15) Ivani, I.; Dans, P. D.; Noy, A.; Pérez, A.; Faustino, I.; Hospital, A.; Walther, J.; Andrio, P.; Goñi, R.; Balaceanu, A.; et al. Parmbsc1: A Refined Force Field for DNA Simulations. *Nat. Methods* **2016**, *13*, 55–58.

(16) Frisch, M. J.; Trucks, G. W.; Schlegel, H. B.; Scuseria, G. E.; Robb, M. A.; Cheeseman, J. R.; Scalmani, G.; Barone, V.; Petersson, G. A.; Nakatsuji, H.; et al. Gaussian 09, Revision A.02. Gaussian, Inc.: Wallingford CT 2016.

(17) Bayly, C. I.; Cieplak, P.; Cornell, W.; Kollman, P. A. A Well-Behaved Electrostatic Potential Based Method Using Charge Restraints for Deriving Atomic Charges: The RESP Model. *J. Phys. Chem.* **1993**, *97*, 10269–10280.

(18) Cieplak, P.; Cornell, W. D.; Bayly, C.; Kollman, P. A. Application of the Multimolecule and Multiconformational RESP Methodology to Biopolymers: Charge Derivation for DNA, RNA, and Proteins. *J. Comput. Chem.* **1995**, *16*, 1357–1377.

(19) Wang, J.; Wang, W.; Kollman, P. A.; Case, D. A. Automatic Atom Type and Bond Type Perception in Molecular Mechanical Calculations. *J. Mol. Graph. Model.* **2006**, *25*, 247–260.

(20) Geronimo, I.; Vidossich, P.; De Vivo, M. Local Structural Dynamics at the Metal-Centered Catalytic Site of Polymerases Is Critical for Fidelity. *ACS Catal.* **2021**, *11*, 14110–14121.

(21) Becke, A. D. Density-functional Thermochemistry. III. The Role of Exact Exchange. *J. Chem. Phys.* **1993**, *98*, 5648–5652.

(22) Lee, C.; Yang, W.; Parr, R. G. Development of the Colle-Salvetti Correlation-Energy Formula into a Functional of the Electron Density. *Phys. Rev. B* **1988**, *37*, 785–789.

(23) Maier, J. A.; Martinez, C.; Kasavajhala, K.; Wickstrom, L.; Hauser, K. E.; Simmerling, C. ff14SB: Improving the Accuracy of Protein Side Chain and Backbone Parameters from ff99SB. *J. Chem. Theory Comput.* **2015**, *11*, 3696–3713.

(24) Dal Peraro, M.; Spiegel, K.; Lamoureux, G.; De Vivo, M.; DeGrado, W. F.; Klein, M. L. Modeling the Charge Distribution at Metal Sites in Proteins for Molecular Dynamics Simulations. *J. Struct. Biol.* **2007**, *157*, 444–453.

(25) Allnér, O.; Nilsson, L.; Villa, A. Magnesium Ion–Water Coordination and Exchange in Biomolecular Simulations. *J. Chem. Theory Comput.* **2012**, *8*, 1493–1502.

(26) R.E.DD.B. https://upjv.q4md-forcefieldtools.org/REDDB (accessed Mar 16, 2020).

(27) Pastor, R. W.; Brooks, B. R.; Szabo, A. An Analysis of the Accuracy of Langevin and Molecular Dynamics Algorithms. *Mol. Phys.* **1988**, *65*, 1409–1419.

(28) Berendsen, H. J. C.; Postma, J. P. M.; van Gunsteren, W. F.; DiNola, A.; Haak, J. R. Molecular Dynamics with Coupling to an External Bath. *J. Chem. Phys.* **1984**, *81*, 3684–3690.

(29) Parrinello, M.; Rahman, A. Polymorphic Transitions in Single Crystals: A New Molecular Dynamics Method. *J. Appl. Phys.* **1981**, *52*, 7182–7190.

(30) Essmann, U.; Perera, L.; Berkowitz, M. L.; Darden, T.; Lee, H.; Pedersen, L. G. A Smooth Particle Mesh Ewald Method. *J. Chem. Phys.* **1995**, *103*, 8577–8593.

(31) Hess, B.; Bekker, H.; Berendsen, H. J. C.; Fraaije, J. G. E. M. LINCS: A Linear Constraint Solver for Molecular Simulations. *J. Comput. Chem.* **1997**, *18*, 1463–1472.
